# Means to Facilitate the Overcoming of Gastric Juice Barrier by a Therapeutic Staphylococcal Bacteriophage A5/80

**DOI:** 10.3389/fmicb.2017.00467

**Published:** 2017-03-23

**Authors:** Ryszard Międzybrodzki, Marlena Kłak, Ewa Jończyk-Matysiak, Barbara Bubak, Anna Wójcik, Marta Kaszowska, Beata Weber-Dąbrowska, Małgorzata Łobocka, Andrzej Górski

**Affiliations:** ^1^Bacteriophage Laboratory, Hirszfeld Institute of Immunology and Experimental Therapy, Polish Academy of SciencesWroclaw, Poland; ^2^Phage Therapy Unit, Hirszfeld Institute of Immunology and Experimental Therapy, Polish Academy of SciencesWroclaw, Poland; ^3^Department of Clinical Immunology, Transplantation Institute, Medical University of WarsawWarsaw, Poland; ^4^Research and Development Center, Regional Specialized HospitalWrocław, Poland; ^5^Laboratory of Microbial Immunochemistry and Vaccines, Hirszfeld Institute of Immunology and Experimental Therapy, Polish Academy of SciencesWroclaw, Poland; ^6^Autonomous Department of Microbial Biology, Faculty of Agriculture and Biology, Warsaw University of Life Sciences – SGGWWarsaw, Poland; ^7^Department of Microbial Biochemistry, Institute of Biochemistry and Biophysics, Polish Academy of SciencesWarsaw, Poland

**Keywords:** bacteriophage, oral administration, gastric juice barrier, phage translocation, antacids, yogurt

## Abstract

In this article we compare the efficacy of different pharmacological agents (ranitidine, and omeprazole) to support phage transit from stomach to distal portions of the gastrointestinal tract in rats. We show that a temporal modification of environment in the animal stomach may protect Twort-like therapeutic antistaphylococcal phage A5/80 (from bacteriophage collection of the Hirszfeld Institute of Immunology and Experimental Therapy PAS in Wroclaw, Poland) from the inactivation by gastric juice effectively enough to enable a significant fraction of orally administered A5/80 to pass to the intestine. Interestingly, we found that yogurt may be a relatively strong in enhancing phage transit. Given the immunomodulating activities of phages our data may suggest that phages and yogurt can act synergistically in mediating their probiotic activities and enhancing the effectiveness of oral phage therapy. We also demonstrate that orally applied phages of similar size, morphology, and sensitivity to acidic environment may differ in their translocation into the bloodstream. This was evident in mice in which a therapeutic staphylococcal phage A5/80 reached the blood upon oral administration combined with antacid agent whilst T4 phage was not detected even when applied in 10^3^ times higher dose. Our findings also suggest that phage penetration from digestive tract to the blood may be species-specific.

## Introduction

Growing antibiotic resistance of bacteria has rejuvenated the interest in using bacteriophages as potential alternatives to antibiotics in the treatment of bacterial infections (Górski et al., 2016). Due to their unique mechanism of antibacterial action phages are able to combat bacteria irrespectively to bacterial antibiotic resistance profiles (Chan et al., 2013, 2016; Cao et al., 2015; Oduor et al., 2016; Ozkan et al., 2016). Phages use specific receptors at the surface of bacterial cells to recognize targeted bacteria (Rakhuba et al., 2010). Therefore their particular feature is a limited spectrum of bacterial hosts. This ensures the targeting of infecting pathogen and saving beneficial saprophytic flora – an important advantage of phages over antibiotics in the fight with bacterial infections (Abhilash et al., 2008). Despite of the progress in knowledge on the potential application of phage therapy in medical and veterinary practice many basic issues still need to be solved. A crucial factor ensuring the effectiveness of phage therapy is the bioavailability of phages at the infection site. With the exception of external infections or infections in easy accessible body cavities it requires the transit of phage from the site of application to the site where the infecting bacteria reside.

The classification of the phages according to morphology and nucleic acid by the International Committee on Taxonomy of Viruses (ICTV) suggest that differences between them may be extensive. Additionally, even phages belonging to the same genera may differ significantly with respect to their sensitivity to different environmental conditions (Jończyk et al., 2011). However, there is a lack of complex studies which compare bioavailability of different phages (Ackermann, 2003; Leiman et al., 2003; Dąbrowska et al., 2005). Detailed knowledge of these processes (chance for the phage penetration to a site of infection) have significant impact in determining therapeutic recommendations for bacteriophage use. Phage bioavailability after oral administration is one of them.

Oral drug application is one of the most convenient for patients, and it was used for phage application by some clinical centers conducting experimental phage therapy (Weber-Dąbrowska et al., 1987; Chanishvili, 2012; Międzybrodzki et al., 2012; Sarker and Brüssow, 2016). It may be used both as a local application for treatment of intestinal infections as well as a systemic administration for treatment of diseases located outside of the digestive tract (Zelasko et al., 2017). However, the data confirming the possibility of the phage transit through gastrointestinal mucosa in humans are scarce (Weber-Dąbrowska et al., 1987; Pagava et al., 2012).

Results of studies on animals concern single phages and indicate that significant differences between phage ability to penetrate the intestinal wall are possible (Dąbrowska et al., 2005). Geier et al. (1973) studied organ penetration of phage λ in mice. They observed that after oral application of 2 × 10^12^ phage particles the titer of phage in blood and organs was a few order of magnitude lower than that after intramuscular, intravenous, or intraperitoneal administration, and that the phage could be detected for a shorter time (up to 30 h after *p.o.* administration versus over 50 h after *i.v.* administration). Keller and Engley (1958) showed that after oral administration of 4 × 10^9^ pfu of *Bacillus megaterium 899a* phage to mice the phage could be detected in blood of most of the mice after 5 min and in urine after 30 min. However, they could not confirm the presence of the phage in blood in 14% of mice. Hildebrand and Wolochow (1962) observed that orally administered T1 phage could penetrate into the lymphatic system as it was shown for some bacteria and large proteins but only in a small percentage of tested animals it was able to penetrate into the blood.

Common sensitivity of phages to an acidic environment may significantly reduce phage titers in stomach and the use of antacid during oral phage therapy seems to be convincing solution of this problem (Dąbrowska et al., 2005; Jończyk et al., 2011). The median gastric pH in fasting humans is 1.7 whereas in further parts of alimentary tract it is over 6 (Dressman et al., 1990). This forms physiological barrier to infection but it may also efficiently inactivate certain phages (Jończyk et al., 2011). Therefore the gastric juice barrier is a key factor which may influence phage bioavailability in further parts of digestive tract as well as in blood and body organs.

Oral phage administration is considered as a possible therapeutic option in experimental phage therapy conducted in patients of the Phage Therapy Unit of the HIIET PAS in Wrocław. One of the most frequently used phages is broadly polyvalent *Staphylococcus aureus* phage A5/80 (designated as vB_SauM_A5/80 according to the recommended nomenclature; Kropinski et al., 2009). Its clone of sequenced genome is known as A5W. This tailed phage belongs to the *Kayvirus* genus of the *Spounavirinae* subfamily of myoviruses that was recently separated by ICTV from the previous *Twortlikevirus* genus (Łobocka et al., 2012).^1^ In addition to A5/80 the *Kayvirus* genus groups at least 11 phages of highly homologous genomic sequences. A few of this phages, A5/80 among them, have been successfully used in the treatment of staphylococcal infections in humans and animals (Gill et al., 2006; Międzybrodzki et al., 2012; Rose et al., 2014). The A5/80 virion consists of an icosahedral capsid (71.5 nm in diameter which is packed with 146-bp dsDNA molecule, and a 214.5 nm long, contractile tail. It was well characterized at the level of genomic sequence (Łobocka et al., 2012), but its bioavailability after oral administration have not been systematically studied. Therefore, our aim was to verify the effectiveness of different methods of neutralization or reducing stomach juice acidity in overcoming the gastric juice barrier by A5/80 bacteriophage and to test if the application of these methods may improve the transfer of A5/80 through mucosa of the gastrointestinal tract into blood in animal model. *Escherichia coli* model phage T4 was used for comparative purposes in these experiments. It is also a representative of *Myoviridae* family of tailed phages (Leiman et al., 2003). Additionally, its total virion length and head diameter (215 and 85 nm, respectively) are close to those of A5/80.

## Materials and Methods

### Animals

Experiments were performed on female Wistar rats (Wroclaw Medical University) and DBA/1LacJ mice (animal facility of the HIIET PAS under the license from the Jackson Laboratory, USA) housed under standard conditions with food and water *ad libitum*. All experiments were approved by the II Local Ethics Committee in Wrocław, Poland. All oral applications in animals were done into the stomach using a curved feeding needle (Kent Scientific, Torrington, CT, USA).

### Phages

T4 phage was purchased from the American Type Culture Collection (ATTC, Rockville, MD, USA). A5/80 phage was obtained from the HIIET PAS therapeutic bacteriophage collection.

Crude phage lysates were prepared according to the modified method of Ślopek et al. (1983). Briefly, phages and the host bacteria were added to peptone water and incubated at 37°C until complete lysis occurred (3–6 h). Phage A5/80 was incubated with cells of *Staphylococcus aureus* 80, and phage T4 was incubated with cells of *E. coli* B strain (standard bacterial hosts for the propagation these phages). Then the suspensions were filtered through a 0.22-μm Millipore filter. Both bacterial strains were from the Polish Collection of Microorganisms (HIIET PAS, Poland).

### Testing the Influence of pH on the Phage Survival

Phage-containing lysate (100 μl, 10^7^ pfu/ml) was added to 900 μl of saline solutions of different pH: 1.1, 2.0, 3.0, 4.0, 5.0, 6.0, 7.0, 7.4 (phosphate buffered saline – PBS), and 9.2. Phages were incubated in solutions at 37°C for 60 min. After incubation, each phage suspension was serially 10-fold diluted with PBS for the determination of phage titer.

### Testing the Influence of Different Factors Neutralizing or Reducing Stomach Juice Acidity on the Phage Survival *In vitro*

Phage-containing lysate (0.5 ml, 10^7^ pfu/ml) was added to 2.0 ml of dihydroxyaluminum sodium carbonate suspension (Alugastrin^®^, Polfa Łódź SA, Poland), ranitidine hydrochloride syrup (Ranitydyna syrop, 75 mg/5 ml, Sanofi-Syntelabo Sp. z o.o., Poland), 3.2% fat milk (OSM Łowicz, Poland), natural yogurt (Danone Sp. z o.o., Poland) or peptone water as control. Samples were prepared in duplicate and incubated at 37°C for 30 min. After incubation, each phage suspension in buffer was serially 10-fold diluted with peptone water for determination of the phage titer. The change of phage activity was calculated as a percentage of the mean phage titer in both samples of each tested factor relative to the mean phage titer in control samples.

### Protocol for Testing the Influence of Different Agents on Overcoming the Gastric Juice Barrier by the Phage

Phage-containing lysate (0.5 ml, 10^7^ pfu/ml) was administered orally to rats deprived of food for 24 h before beginning of the experiment. Before phage administration, the rats were given:

- an oral dose of 1.0 ml of 68 mg/ml dihydroxyaluminum sodium carbonate suspension (Alugastrin^®^, Polfa Łódź SA, Poland) 5–30 min. before phage administration,
- 5–75 mg/kg body weight oral dose of ranitidine hydrochloride syrup (Ranitydyna syrop, 75 mg/5 ml, Sanofi-Syntelabo Sp. z o.o., Poland) 2 h before phage administration,
- 2–50 mg/kg body weight of intraperitoneal ranitidine (Zantac^®^, for injections, 25 mg/ml, GlaxoSmithKline Export Ltd, United Kingdom) 2 h before phage administration,
- 2.5–10 mg/kg body weight of intraperitoneal omeprazole (Losec^®^, for injections, 4 mg/ml, AstraZeneca AB, Sweden) 2 h before phage administration,
- 2.0 ml of 3.2% fat milk *per os* (OSM Łowicz, Poland) 1 or10 min. before phage administration,
- 2.0 ml of natural yogurt (Danone Sp. z o.o., Poland) *per os* 1 or 10 min. before phage administration.

Control animals were given the phage lysates only. Rats were sacrificed 30 min. after phage administration, and fragments of duodenum, the middle section of the small intestine, and the caecum were collected.

### Testing the Influence of Alugastrin on Gastrointestinal Transit of A5/80 Phage in Rats

Rats were deprived of food for 24 h before beginning of the experiment. They were administered 1.0 ml of Alugastrin *per os* 1–60 min prior to A5/80 phage lysate application (dose: 0.5 ml, 10^8^ pfu/ml). They were sacrificed 5–120 min after phage administration and the intestinal contents were collected to determine the phage titer.

### Testing the Bioavailability of the Phages after Administration to Rats

Rats were deprived of food for 24 h before beginning of the experiment. In the first experiment A5/80 phage lysate (1.0 ml, 2 × 10^9^ pfu) was applied orally 15 min after Alugastrin (1.0 ml orally or intravenously) and the rats were sacrificed for collection of blood and liver samples before experiment (control), and 1, 2, 4, and 18 h after. In the second experiment Alugastrin was applied 10 min before phage administration (6 × 10^7^ pfu of A5/80 phage, and 4 × 10^7^ pfu of T4 phage in 0.5 ml of the phage lysate) and samples of blood, lymph (from *cisterna chyli*), mesenteric and thoracic lymph nodes, and the middle section of the small intestine were collected. To visualize the lymph to enable its collection the animals received *per os* 1 ml of rape oil 15 min before euthanasia according to Hildebrand and Wolochow (1962). The animals were sacrificed 30 min after phage administration and samples of blood, lymph, mesenteric and thoracic lymph nodes, and small intestine (middle part) were collected to determine the phage titer.

### Testing the Influence of Alugastrin on Bioavailability of the Phages after Oral Administration to Mice

The mice were given 0.2 ml of the appropriate phage lysates. Ten minutes before phage administration, they were given an oral dose (0.2 ml) of Alugastrin. Control animals were given the phage lysates only. After 1 h they were sacrificed and heparinized whole blood samples and liver fragments were collected for determination of the phage titer.

### Determination of the Phage Titers in Samples

The phage titers both in *in vitro* as well as *in vivo* experiments were determined in duplicate samples using the double-layer agar method according to Adams (1959). Cells of *E. coli* B strain and cells of *S. aureus* 80 strain that were used for the T4 and A5/80 phage propagation were used as indicator strain, respectively. Ten centimeter fragments of duodenum, the middle section of the small intestine were rinsed out with 5 ml of PBS for phage titration in diluted intestine contents. The content of caecum was diluted in PBS in proportion of 100 mg/1 ml. Tissue fragments (spleen, liver, lungs, kidney, brain, and lymph nodes) were homogenized in PBS in proportion of 100 mg tissue per 1 ml of PBS. Lymph was diluted 10–40 times with PBS for phage titer determination. The phage titer in blood was assayed in undiluted blood samples or in samples diluted with PBS when titer was high.

### Statistical Analysis

Results are presented as mean phage titer (± standard error of the mean, SE) in the analyzed sample. Differences between the study groups were compared to the control using the non-parametric Mann–Whitney *U*-test (in cases where the number of mice in compared groups was 4 or higher). The differences between the means were considered statistically significant at *p* < 0.05.

## Results

**FIGURE 1:**
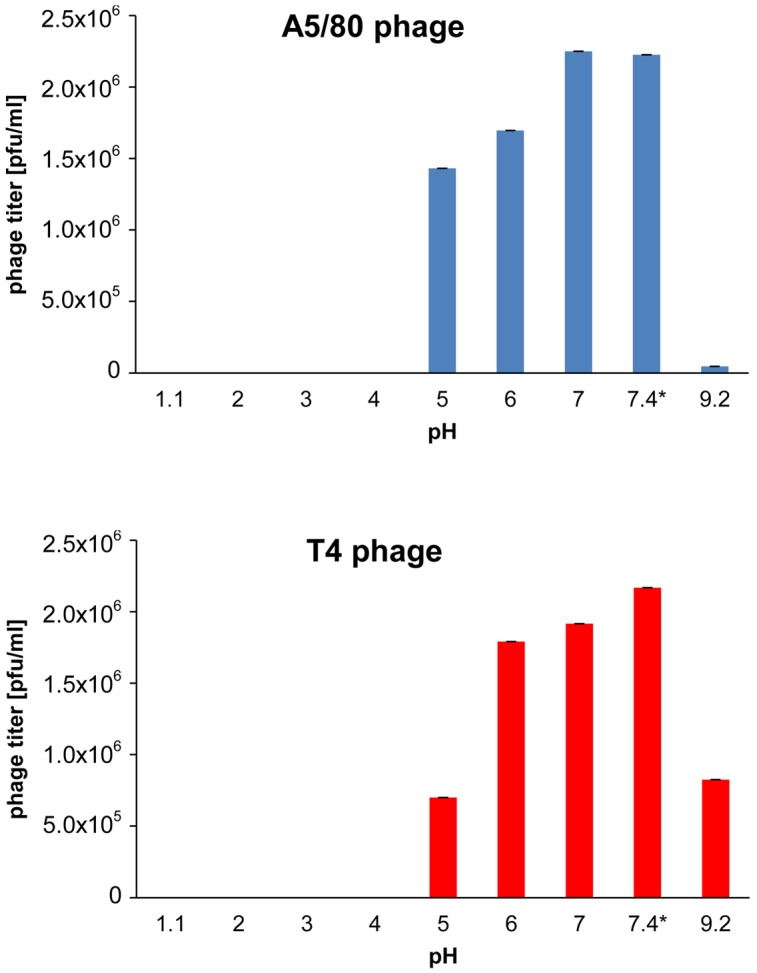
**Effect of acidity on phage survival**. ^∗^Phosphate buffered saline (pH 7.4) was used as control. The phages were incubated in buffers at 37°C for 60 min.

Both, A5/80 and T4, phages used in this study appeared to be sensitive to inactivation at pH below 5, despite, that they differed in the sensitivity to alkaline environment (**Figure 1**). Thus, one can expect that they will be inactivated in a stomach, upon exposure to gastric acid.

Commonly used pharmaceuticals that neutralize the acidity of stomach juice or inhibit its production, when administered orally are dihydroxyaluminum sodium carbonate (Alugastrin), ranitidine hydrochloride (a H_2_-receptor antagonist) or omeprazole (a proton pump inhibitor). Additionally, drinking of milk or fermented milk products has been considered as a natural stomach ulcer protective therapy (see e.g., Ippoliti et al., 1976; Modlin, 1995; Elmståhl et al., 1998). Thus, we tested whether certain of these various acidity decreasing agents will not inactivate A5/80 or T4 bacteriophage *in vitro*, when added to the suspensions of these phages (**Table 1**). Milk and yogurt had a negligible effect on the titer of both phages. The influence of Alugastrin on the titer of phage T4 was also negligible, but the titer of phage A5/80 decreased to 40% after 30 min incubation with Alugastrin. Ranitidine hydrochloride syrup decreased the titer of both phages to about 2% or less.

**Table 1:** Comparison of the bioavailability of A5/80 phage at different time points after its oral versus intravenous administration to rats.

Time since the phage administration	Phage titer after oral administration [pfu/ml]				Phage titer after intravenous administration [pfu/ml]			
	Blood		Liver		Blood		Liver	
	*n*	Mean	*n*	Mean	*n*	Mean	**n**	Mean
1 h	2	0	2	0	3	485^†^	2	419^‡^
2 h	3	0	2	0	1	50	–	–
4 h	2	0	2	0	1	150	1	33
18 h	2	0	2	0	1	5	1	5

*All animals received phage at dose of 2 × 10^*9*^ pfu (in 1.0 ml of phage lysate). Alugastrin (1.0 ml) was applied 15 min before the phage administration. ^†^, min.-max. value: 405–555 pfu. ^‡^, min.-max. value: 373–465 pfu. No phage was detected neither in blood nor liver in a control group of two animals (non-treated with the phage). N, number of tested animals*.

**FIGURE 2:**
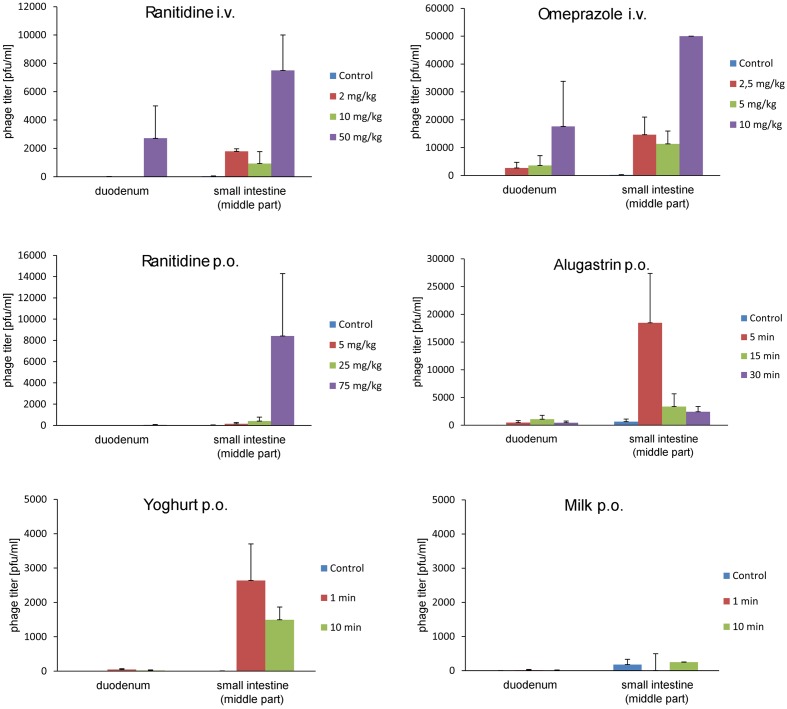
**Influence of ranitidine, omeprazole, Alugastrin, yogurt, and milk on A5/80 phage ability to survive in the stomach and to pass into the small intestine**. Samples for phage titer determination were taken 30 min after oral administration of 0.5 ml of phage lysate (10^7^ pfu/ml). Groups of animals differed depending on a dose of stomach acid inhibitor (ranitidine or omeprazole), or time of the oral application of potential stomach juice neutralizer (1.0 ml of Alugastrin, 2.0 ml of yogurt, or 2.0 ml of milk) before phage administration. Control animals were given the phage lysates only. Shown is mean phage titer ± SE in analyzed samples (*n* = 2–6).

We tested whether the aforementioned agents can increase the ability of A5/80 phage to overcome the inactivating barrier of stomach juice *in vivo* (**Figure 2**). Oral Alugastrin and ranitidine (oral or parenteral) as well as parenteral omeprazole strongly increased the ability of A5/80 phage to overcome gastric juice barrier and pass to the further parts of the gastrointestinal tract in rats. The effect of the highest applied oral versus intravenous dose of ranitidine (75 mg/kg and 50 mg/kg, respectively) on phage titer attained in a small intestine was comparable but more phages were detected in duodenum after *i.v.* application, although a 33% lower dose of the H_2_ inhibitor was used. When lower doses of ranitidine were applied we also observed higher phage transit into the intestine in case of intravenous administration (the effect of the lowest *i.v*. dose, 2 mg/kg, was almost 50 times higher than effect of the lowest oral dose – 5 mg/ml). The activity of omeprazole was much higher. When this proton pump inhibitor was applied at a dose of 10 mg/kg it increased the phage titer in a small intestine over six times more as compared to the effect of 50 mg/kg of intravenous ranitidine. At a dose of 2.5 mg/kg the effect of omeprazole was over 8 times stronger than *i.v*. ranitidine at a dose of 2 or 10 mg/kg. When rats were pretreated with 1.0 ml of Alugastrin, the highest phage titer was observed in the small intestine when the phage was applied 5 min. after the administration of Alugastrin and it was over two times higher than the effect of oral ranitidine at a dose of 75 mg/kg. Milk did not improve the intestinal transit of the A5/80 phage significantly (**Figure 2**). Surprisingly, yogurt used just 1 min before the application of the phage increased its titer in the small intestine six times more than the medium dose of oral ranitidine (25 mg/kg). Phage titers in the caecum were usually much lower than those in a small intestine (data not shown).

**FIGURE 3:**
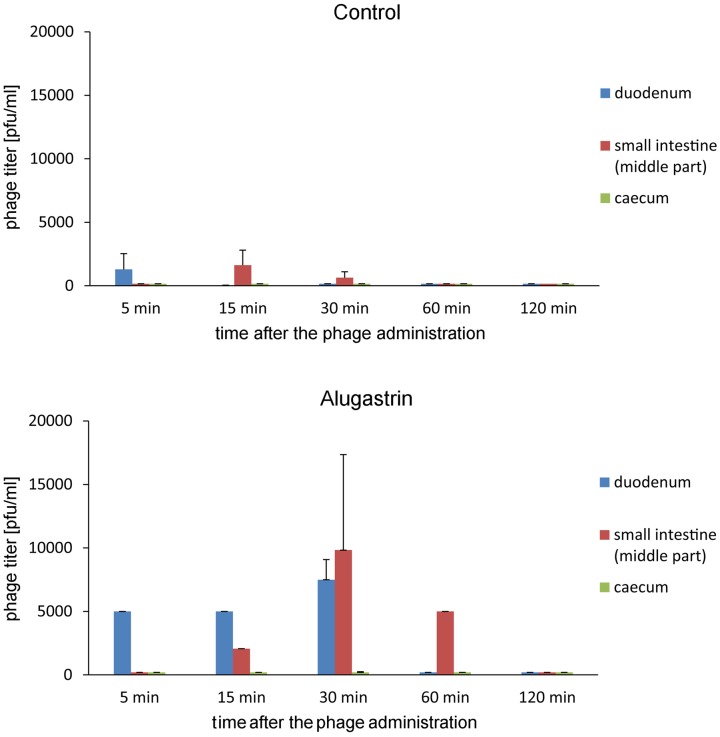
**Influence of Alugastrin on A5/80 phage intestinal transit. 1.0 ml of antacid was given to animals 15 min before administration of the phage**. Samples of the intestinal contents were collected for phage titer determination at different time points (5–120 min) after oral administration of 0.5 ml of phage lysate (10^8^ pfu/ml). Shown is mean phage titer ± SE in analyzed samples (*n* = 1–4).

**FIGURE 4:**
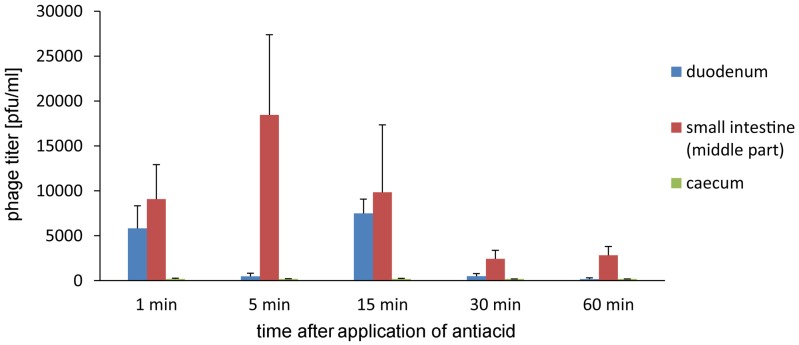
**Intestinal transit of A5/80 phage applied orally at different time points (1–60 min) after oral administration of an antacid (1 ml of Alugastrin)**. Samples of the intestinal contents were collected for phage titer determination 30 min after administration of 0.5 ml of phage lysate (10^8^ pfu/ml). Shown is mean phage titer ± SE in analyzed samples (*n* = 3–4).

Further experiments on the influence of Alugastrin on bioavailability of A5/80 phage in gastrointestinal tract of rats showed that the phage reached highest titer in the duodenum and small intestine 30 min after its application (**Figure 3**), and that the optimal time for administration of Alugastrin to increase the phage titer in these parts of gastrointestinal tract is its use 1–15 min before the phage administration (**Figure 4**).

We were not able to confirm the presence of active A5/80 phage in blood samples collected during all the above experiments (data not shown). Therefore we conducted more detailed experiments on A5/80 bioavailability after oral administration in rats (**Tables 1**, **2**). Our experiment in which we used a 40 times higher phage dose also did not confirm phage penetration through rat intestinal mucosa into the bloodstream (**Table 1**). Moreover we could not confirm that A5/80 phage was able to penetrate into lymph (**Table 2**). The same results were obtained for T4 phage. Unexpectedly, bioavailability studies done in mice showed contrasting results (**Table 3**). They confirmed that the A5/80 phage was able to penetrate into the bloodstream of mice after its oral administration but it required concomitant use of an antacid (the highest phage titer in blood was observed 60 min after the phage application). In contrast, T4 phage applied at a dose 1000 times higher than A5/80 was detected in blood only in trace amounts (even when the administration of phage was preceded by the administration of Alugastrin).

**Table 2:** Orally administered A5/80 and T4 phage penetration into blood, lymph, mesenteric and thoracic lymph nodes, and small intestine (middle part) in rats.

Phage/sample	Phage titer in analyzed samples [pfu/ml]								
	Control			Phage			Phage+Alugastrin		
	*n*	Mean	*SE*	*n*	Mean	*SE*	*n*	Mean	*SE*
A5/80 phage									
Blood	4	0	0	4	0	0	4	0	0
Lymph	4	0	0	4	0	0	4	0	0
Mesenteric lymph nodes	4	0	0	4	0	0	4	0	0
Thoracic lymph nodes	4	0	0	4	0	0	4	0	0
Small intestine	4	0	0	4	37	37	4	444	271
T4 phage									
Blood	3	0	0	3	0	0	3	0	0
Lymph	2	0	0	2	0	0	1	0	–
Mesenteric lymph nodes	3	0	0	3	0	0	3	6	5
Thoracic lymph nodes	2	0	0	3	0	0	3	5	3
Small intestine	3	12	12	3	1 348	1 116	3	14 667	12 719

*Alugastrin was applied 10 min before the phage administration. Animals were euthanized 30 min after application of the phage preparation (0.5 ml per animal). The phage dose was: 6 ×*10*^*7*^ pfu of A5/80 phage, and 4 ×*10*^*7*^ pfu of T4 phage. 15 min before termination of the experiment all rats (including the control group) received *per os* 1 ml of rape oil to visualize the lymph. SE, standard error of the mean; n, number of tested animals*.

**Table 3:** Bioavailability of A5/80 and T4 phages in blood and liver after their oral administration to mice.

Phage	Dose [pfu]	Group	Time from the phage application	Phage titer in samples [pfu/ml]					
				Blood			Liver		
				*n*	Mean	*SE*	*n*	Mean	*SE*
A5/80	5 × 10^6^	Control	15 min	5	217	212	5	20	20
			30 min	6	1	1	6	0	0
			60 min	4	17	17	4	2	2
			120 min	4	11	11	4	1	1
			180 min	4	21	19	4	0	0
		Alugastrin	15 min	4	108	106	4	3	1
			30 min	9	806	482	9	155^∗^	70
			60 min	7	2 783^∗^	936	7	637^∗^	463
			120 min	4	363	363	4	33	32
			180 min	4	28	28	4	10	6
T4	5 × 10^6^	Control	60 min	3	10	8	3	0	0
		Alugastrin	60 min	3	3^∗∗^	1	3	0^∗∗^	0
	7 × 10^9^	Control	60 min	3	1	1	3	3	1
		Alugastrin	60 min	3	18^∗∗^	15	3	66	63

*Alugastrin (0.2 ml) was applied 10 min before the phage administration. Control group received only 0.2 ml of appropriate phage lysate. SE, standard error of the mean; n, number of tested animals. *^∗^p* < 0.05 when compared to control group. *^∗∗^p* < 0.05 when compared to A5/80 phage and Alugastrin treated group tested after 60 min*.

## Discussion

We show here that a temporal modification of environment in the animal stomach may protect therapeutic antistaphylococcal phage A5/80 from the inactivation by gastric juice effectively enough to enable a significant fraction of orally administered A5/80 to pass to the intestine and even to a bloodstream. Our results comparing the efficacy of different pharmacological agents in protecting phage against inactivation by gastric juice revealed that a proton pomp inhibitor, omeprazole was the most efficient followed by a H_2_ receptor antagonist, ranitidine, and dihydroxyaluminum sodium carbonate – an agent that is traditionally used in phage treatment conducted at the Phage Therapy Unit in Wroclaw (Międzybrodzki et al., 2012). The data showing the applicability of omeprazole and ranitidine in promoting phage transit to intestinal lumen may be relevant in patients with gastrointestinal disorders where acid production should be under strict control (e.g., peptic inflammation and ulcer).

Although *in vitro* A5/80 phage is nearly completely inactivated upon incubation with hyperosmotic ranitidine syrup or, to a lesser extent, with alkaline Alugastrin (its pH is between 9.9 and 10.2 in a 1:25 suspension^2^), *in vivo* the administration of ranitidine or Alugastrin to animals prior to the oral phage administration significantly increased the number of phages that could pass the stomach juice barrier retaining their activity. A time period separating the administration of ranitidine or Alugastrin from the administration of phage suspension appeared to be an important parameter influencing the phage-protective activity of each of these pharmaceuticals (**Figure 2**), which should be taken into consideration in planning the phage therapy regime whenever phage has to be administered *per os*. To our surprise, yogurt turned out to be relatively efficient in protecting phage from stomach juice, despite that in the case of milk no clear effect was observed. This may be associated with mildly acidic pH of Danone natural yogurt (5.29 according to Çaglar et al., 2011) and with the yogurt buffering activity (Kargul et al., 2007). In *in vivo* studies, the ingestion of yogurt was shown to stabilize the gastric pH for 1 h at the level exceeding 3.5 (Martini et al., 1987). In contrast to that whole milk as well as low-fat milk could cause the increased gastric acid secretion, despite its transient buffering activity (Ippoliti et al., 1976; Khanna and Abraham, 1990; Marotta and Floch, 1991). Taken together, our data suggest that yogurt when added to the current therapeutic protocols of oral phage administration might improve the effectiveness of therapy. This may also have important and therapeutic implications related to suggested immunomodulating activities of phages which may contribute to immunological hemostasis in intestines referred to as probiotic like action of phages (Górski and Weber-Dąbrowska, 2005). Given the well-known probiotic activities of yogurt in gut (reviewed by Adolfsson et al., 2004) and the present data pointing to the so far unknown ability of yogurt to promote phage survival and gastrointestinal passage it suggest that phages and yogurt might act synergistically in mediating their probiotic activities and enhancing the effectiveness of oral phage therapy, for example in the treatment of digestive tract infections. Taking under consideration recent data presented by Przybylski et al. (2015) on the anti-adenoviral activity of T4 phage one may speculate that it might include not only bacterial but also viral infections. Potential therapeutic value of this approach requires further studies.

We also aimed to check if overcoming gastric juice barrier by A5/80 phage enables its systemic detection upon oral administration. Because we were not able to detect orally administered A5/80 as well as T4 phage (used here for comparative purposes) in the bloodstream of rats, even when the administration of these phages was combined with antacid agent we performed experiments on mice. Unexpectedly, results for A5/80 and T4 were completely different, despite the similarities in the size and morphology of these phages and their similar sensitivity to acidic environment *in vitro*. This was evident where a staphylococcal A5/80 phage reached the blood upon oral administration combined with gastric acid neutralization whilst T4 phage did not, even though it was applied in 1000-times higher dose. At least a few phage features and non-phage dependent factors could possibly cause this difference. The first one is phage susceptibility to digestive enzymes and bile salts (Ma et al., 2008). However, a sensitivity to these digestive tract components is unlikely in the case of T4 because T4 was able to survive in the small intestine of rats even better than A5/80 (**Table 2**). The second one is a possible phage interaction with bacteria of gut microbiome of tested animals. The A5/80 or T4 adsorption to dead bacteria or to the remnants of their envelopes containing phage receptors will inevitably lead to the irreversible phage inactivation. The adsorption to living bacteria can have several outcomes, with the exception of lysogeny and transduction, as A5/80 and T4 are obligatorily virulent and non-transducing phages (Łobocka et al., 2012, 2014; and references therein). It can be productive leading to a temporal decrease and later to the increase in phage titer, when the progeny of adsorbed phages is released from the infected bacteria. Alternatively, it can be non-productive due to the inability of infected cell to support phage development, to the degradation of injected phage DNA by bacterial restriction enzymes or the CRISPR-Cas immunity system, to the abortive infection mechanisms, or to the bacterial toxin-antitoxin system activation (reviewed by Labrie et al., 2010). In our experiments the decrease of A5/80 or T4 titer upon 15 and 45 min. incubation of each of these phages with the content of mice or rat intestine, did not exceed one order of magnitude (Supplementary Tables 4–6). Moreover, in the case of certain mice we observed an increase in T4 phage titer 45 min. after the phage incubation with the intestine content. These changes are indicative of both the possibility of inactivation of some A5/80 or T4 phages in the intestine as well as the possibility of productive infection of some intestinal bacteria by T4. However, in our opinion they are too small to explain the difference in murine blood titers of A5/80 and T4 applied in 10^3^ higher dose than A5/80. We were able to detect bacterial strains susceptible to both phages in the rat intestines (detailed data presented in Supplementary Materials), but were not able to detect them in the intestine of mice. Conceivably, in mice they do not predominate among enterobacteria of the gut microbiome. Another reason of the absence of T4 in blood could be its direct inactivation in blood – for example by the presence of anti-T4 phage or cross-reacting antibodies (Dąbrowska et al., 2014). Our control experiments showed that 60 min incubation of T4 phage in full blood samples of mice (at 37°C) did not decreased its activity, and that the phage incubation with rat or murine serum resulted only in less than 25% drop in its titer (for details please see Supplementary Tables 2, 3). Therefore, we hypothesize that differences in the ability of A5/80 and T4 to penetrate from the intestine to blood in our experiments might result from different interactions of these phages with intestinal mucus layer and/or intestinal mucosa.

T4-like phages are natural components of mammalian gut as indicated by several cases of their isolation from stool samples (see e.g., Furuse et al., 1983; Kutter et al., 1995; Chibani-Chennoufi et al., 2004). The digestive tract is a natural reservoir of their host bacteria. Evolutionary, these phages could hardly benefit from the passage from the intestinal lumen to a bloodstream. Instead one may expect that they developed strategies to ensure their retention in the digestive tract. The T4 capsid-exposed protein Hoc was shown previously to interact with mammalian organisms (Dąbrowska et al., 2006, 2007) and to bind to mucin glycoproteins (Barr et al., 2013). Recently Barr et al. (2015) demonstrated that the T4 adherence to mucus and Hoc interaction with mucin glycoproteins are responsible for the subdiffusive motion of T4 in the mucus, as compared to the diffusive motion of the T4Δ*hoc* phage. As a result of the subdiffusive motion wild-type T4 could reduce the bacterial colonization of the epithelium 4,000-fold more efficiently than its Δ*hoc* mutant. Possibly, T4 is trapped in the intestinal mucus and thus cannot penetrate further layers of the intestinal barrier. Studies are in progress to find out whether the *hoc* gene deletion will influence the systemic bioavailability of orally administered T4 phage.

In the intestine, a physical barrier between the intestinal lumen, the lamina propria and the mucosal-associated lymphoid tissue is the intestinal epithelium. It is formed by a single layer of cells and mostly contain enterocytes (absorptive epithelial cells), microfold (M) cells (non-absorptive epithelial cells) and goblet cells scattered among them. Mucus secreted by the goblet cells spatially compartmentalizes the bacteria to the lumen (Johansson et al., 2011). The paracellular flux through this layer is limited by tight junctions that form interconnections between the most apical parts of the epithelial cells. The size of particles that can penetrate through tight junctions does not exceed 10 nm (Fihn et al., 2000; Shen et al., 2011), which is too small for the passage of A5/80 and T4 phage. An alternative is a transenterocytic pathway or the M-cell-mediated pathway. The former occurs by endocytosis through the apical enterocyte membrane, followed by intracellular trafficking and exocytosis through the basolateral membrane (reviewed by Yu et al., 2016). However, the endocytosis of A5/80 by enterocytes might require specific receptors as it was shown for certain pathogens that are translocated via this pathway across the intact intestinal barrier. Thus, the more likely way of A5/80 passage through the intestinal barrier is the M-cell-mediated pathway. M cells are specialized in antigen sampling, have a strong transcytotic capacity, and can transport many bacteria and viruses, as well as other antigens from the intestinal lumen to the underlying lymphoid tissues to induce immune responses (Kyd and Cripps, 2008; Gonzalez-Hernandez et al., 2014; Chamcha et al., 2015). Limitations of this pathway are the low proportion of M cells (1%) in the intestinal epithelium, as compared to other cells, and a possibility of capturing the transported bacteria or viruses by macrophages and dendritic cells (Yu et al., 2016).

Our results demonstrating weak, if any, T4 phage ability to translocate through the intestinal mucosa are in agreement with the results of Bruttin and Brüssow (2005) who were not able to detect T4 phages in blood after their oral application to human volunteers. The possibility of passage of orally administered T4-like coliphages (isolated from stool samples of pediatric patients with diarrhea and from environmental water samples) through the intestinal tract of mice was demonstrated previously, but in all these cases the presence of phages was restricted to a gut lumen (Chibani-Chennoufi et al., 2004). In a limited study using three horses, Letarova et al. (2012) also was not able to detect fecal phages in blood although they were detected in faces of animals even over 10^7^ pfu/ml. Only Majewska et al. (2015) reported detection of T4 phage in blood after its oral application to mice. In our opinion it could be facilitated by a long-term phage application (the experiment lasted for 100 days), a high phage dose (4 × 10^10^ pfu/ml of drinking water), as well as by a repeated collection of blood samples which could cause stress and hence could also influence the permeability of the gastrointestinal tract mucosa.

The recent data by Thannesberger et al. (2017) indicate that phages may be detected in large quantities in human urine which suggest that they could translocate from the intestinal tract and migrate to other tissues which has a clear clinical significance and relation to our current data presented in this article. The data of Weber-Dąbrowska et al. (1987) and Pagava et al. (2012) suggesting that in patients on oral phage therapy phages may translocate from intestines to peripheral blood, as well as our data, confirm the value of oral phage application as efficient means of delivering phages to sites of infections and thereby successful therapy in patients with bacterial infections, as described earlier (Międzybrodzki et al., 2012). Interspecies differences in phage translocation reported in this article suggest that this phenomenon should be studied in detail in human clinical trials.

## Author Contributions

RM: design of the study, performance of experimental part, analysis and interpretation of data for the study, drafting the manuscript (mainly) and revising it critically for important intellectual content, and final approval of the version to be published. MKł, EJ-M, and BB: performance of experiments on animals, drafting the manuscript (partly), and final approval of the version to be published. AW, MKa, and MŁ: performance of some *in vitro* experiments, drafting the manuscript (partly), and final approval of the version to be published. BW-D: production of the phage preparations, drafting the manuscript (partly), and final approval of the version to be published. AG and MŁ: interpretation of data for the study, revising the manuscript critically for important intellectual content, and final approval of the version to be published.

## Conflict of Interest Statement

BW-D, Mł, AG, and RM have filed patent applications for anti-bacterial use of phages. The other authors declare that the research was conducted in the absence of any commercial or financial relationships that could be construed as a potential conflict of interest.

## Footnotes

1 http://ictvonline.org/proposals/2015.005a-gB.A.v3.Kayvirus.pdf

2 http://www.pharmacopeia.cn/v29240/usp29nf24s0_m26270.html
