# Supplementary material for: Means to Facilitate the Overcoming of Gastric Juice Barrier by a Therapeutic Staphylococcal Bacteriophage A5/80
Source: Front Microbiol. 2017 Mar 23;8:467. doi: 10.3389/fmicb.2017.00467 (PMC5362586; doi:10.3389/fmicb.2017.00467)
Supplement: Supplementary file 1 [file Data_Sheet_1.pdf]

Supplementary Material

Means to overcome the gastric juice barrier by a therapeutic staphylococcal bacteriophage A5/80

Ryszard Międzybrodzki*, Marlena Kłak, Ewa Jończyk-Matysiak, Barbara Bubak, Anna Wójcik, Marta Kaszowska, Beata Weber-Dąbrowska, Małgorzata Łobocka, Andrzej Górski[3]

*** Correspondence:** Ryszard Międzybrodzki: mbrodzki@iitd.pan.wroc.pl

1 Supplementary experimental procedures

1.1 Testing the inhibition of phage activity by serum of full blood

Phage lysate (100 µl, 10^7 pfu/ml) was added to 500 µl of serum (rats and mice), full heparinized blood samples (mice), or peptone water as a control. The mixtures were incubated for 60 min at 37°C. After incubation, each sample was serially ten-fold diluted with peptone water for determination of the phage titer using the double-layer agar method according to Adams (1959). All assays were performed in duplicate samples (phage-specific standard bacterial hosts were applied). The inhibition of phage activity was calculated as a percentage of the phage titter in tested sample in comparison to the control.

1.2 Testing the phage adsorption to the content of inestine

To collect intestine content 10-cm fragments of the distal section of the small intestine taken from mice were rinsed out with 5 ml of peptone water or the 0.5 g of content of rat caecum was diluted with 5 ml of peptone water and filtered through a nylon mesh. After centrifugation of 2 ml of each suspension in 15 ml polypropylene conical centrifuge tube (LP Italiana S.p.A., Italy) for 20 min at 700 × *g* (4°C), 200 µl of A5/80 or T4 phage lysate (10^5 pfu/ml) was added to the sediment and incubated at 37°C with periodical gentle shaking. After 15, and 45 min of incubation 100 µl of each suspension was collected, diluted with peptone water up to the final volume of 1.0 ml, and filtered through a 0.22 µm Millipore filter. Then routine test dilution (RTD) for each sample was performed to choose the correct sample dilution for further determination of the phage titer using the double-layer agar method of Adams (1959). All assays were performed in duplicate samples (phage-specific standard bacterial hosts were applied). Phage lysate incubated in peptone water served as a control. Phage activity after the incubation was calculated as a percentage of the mean phage titter for each tested sample in comparison to the mean phage titter in control samples.

1.3 Isolation of bacteria from digestive tract of animals

Ten cm fragments of duodenum, the middle section of the small intestine were rinsed out with 5 ml of peptone water whereas the content of caecum was diluted in peptone water in proportion of 100 mg/1 ml. After filtration through a nylon mesh 100 μl, the suspension was cultured on MacConkey or blood agar plates at 37°C. Up to 4 randomly selected colonies from each plate were used in further tests.

1.4 Phage typing

Bacterial cultures were prepared in liquid broth medium and the suspensions were spread on plates with solid agar medium. Sensitivity of bacterial strains to A5/80 or T4 phages was verified using Routine Test Dilution (RTD).

1.5 MALDI-TOF MS BioTyper identification of bacterial strains

Intact bacterial cells were deposited on a target plate, and analyzed using an using MALDI-TOF Ultraflextreme (Bruker, Germany) instrument with Biotyper software. MALDI-TOF MS spectra were recovered from formic acid/acetonitrile bacterial extracts overlaid with α-cyano-4-hydroxy-cinnamic acid matrix in linear positive mode. Mass spectra obtained from m/z 2000 to 20000 were used for clustering and spectral library searching of *E. coli* strains. Log(score) value above 2.0 which was considered as high confidence identification.

1.6 Testing the influence of Alugastrin on gastrointestinal transit of T4 phage and its blood titer after oral administration to rats

Rats were deprived of food for 24 hours before beginning of the experiment. They were administered 1.0 ml of Alugastrin *per os* 60 min prior to T4 phage lysate application (dose: 0.5 ml, 5.8×10^9 pfu per animal). The rats were sacrificed 60 min after phage administration and the intestinal contents and blood samples were collected to determine the phage titer. Control animals were given the phage lysates only.

1.7 Testing the blood titer of A5/80 phage after its intravenous administration to mice

Mice were administered intravenously 0,2 ml of the A5/80 phage lysate (5.0×10^7 pfu/ml) serially diluted with peptone water. They were sacrificed 60 min later for the collection of blood samples.

2 Supplementary Figures and Tables

2.1 Tables

Supplementary Table 1. Comparison of A5/80 and T4 phage activity after its incubation for 30 min at 37°C with dihydroxyaluminum sodium carbonate suspension, ranitidine hydrochloride syrup, 3.2% fat milk, or natural yoghurt. The changes in phage activities are represented as % changes in the phage titer, using the activity of phage after its incubation in peptone water as a control (100.0%). All samples were tested in duplicate. Experimental procedures were described in details in the main text of the manuscript.

Sample	A5/80 phage activity	T4 phage activity
Dihydroxyaluminum sodium carbonate suspension	40.0%	97.8%
Ranitidine hydrochloride syrup	2.3%	0.6%
3.2% fat milk	90.2%	96.8%
Natural yoghurt	99.3%	89.2%

Supplementary Table 2. The activity of T4 phage after 60 min of incubation at 37°C with serum or full blood samples taken from DBA1/LacJ mice. The changes in phage activities are represented as % changes in the phage titer, using the activity of phage after its incubation in peptone water as a control (100%). All samples were tested in duplicate.

**Mouse No.**	**Phage activity**	
	**serum samples**	**full blood samples**
1.	94.0%	130.1%
2.	54.2%	112.0%
3.	79.5%	108.4%
**Mean**	**75.9%**	**116.9%**

Supplementary Table 3. The activity of T4 phage after 60 min of incubation at 37°C with serum taken from Wistar rats. The changes in phage activities are represented as % changes in the phage titer using the activity of phage after its incubation in peptone water as a control (100.0%). All samples were tested in duplicate.

**Rat No.**	**Phage activity in serum samples**
1.	105.5%
2.	57.5%
3.	63.9%
**Mean**	**75.6%**

Supplementary Table 4. The activity of A5/80 phage after the incubation at 37°C with the intestine content of DBA1/LacJ mice. The changes in phage activities are represented as % changes in the phage titer using the activity of phage after its incubation in peptone water as a control (100%).

Mouse No.	Phage activity	
	after 15 min	after 45 min
1.	59.9%	16.9%
2.	76.9%	39.8%
3.	3.5%	1.3%
**Mean**	**46.6%**	**19.4%**

Supplementary Table 5. The activity T4 phage after the incubation at 37°C with the intestine content of DBA1/LacJ mice. The changes in phage activities are represented as % changes in the phage titer using the activity of phage after its incubation in peptone water as a control (100%).

Mouse No.	Phage activity	
	after 15 min	after 45 min
1.	46.7%	112.0%
2.	5.8%	29.4%
3.	6.6%	109.0%
4.	72.1%	99.3%
**Mean**	**33.0%%**	**87.4%**

Supplementary Table 6. The activity T4 phage after the incubation at 37°C with the caecum content of Wistar rats. The changes in phage activities are represented as % changes in the phage titer using the activity of phage after its incubation in peptone water as a control (100%).

Rat No.	Phage activity	
	after 15 min	after 45 min
1.	47.5%	24.8%
2.	50.0%	96.5%
3.	20.1%	43.3%
**Mean**	**39.2%**	**54.9%**

Supplementary Table 7. Sensitivity of selected bacteria from digestive tract of Wistar rats to T4 or A5/80 phage.

Rat No.	Sample	MacConkey agar culture		Blood agar culture	
		**Bacterial growth**	**Positive T4 phage typing / No. of tested strains**	**Bacterial growth**	**Positive A5/80 phage typing / No. of tested strains**
**1**	Duodenum	-	0/0	-	0/0
	Small intestine (middle part)	++	4/4	++	0/4
	Small intestine (distal part)	++	4/4	++	0/3
	Caecum	+++	4/4	+++	0/3
**2**	Duodenum	-	0/0	-	0/0
	Small intestine (middle part)	++	4/4	++	1/1
	Small intestine (distal part)	++	4/4	++	0/1
	Caecum	++	4/4	++	3/3
**3**	Duodenum	-	0/0	-	0/0
	Small intestine (middle part)	++	4/4	++	1/3
	Small intestine (distal part)	+	4/4	++	2/3
	Caecum	+++	4/4	+++	0/3

Legend: (-) – no bacterial growth; (+) – 1-10 colonies per plate; (++) – 11-100 colonies per plate; (+++) – over100 colonies per plate.

Supplementary Table 8. Sensitivity to T4 phage of selected bacteria from digestive tract of DBA1/LacJ mice, able to grow on MacConkey agar.

Mouse No.	Sample	Colony tested	T4 phage typing	BioTyper identfication[a]
**1**	Doudenum	no growth		
	Small intestine (middle part)	no growth		
	Small intestine (distal part)	A	negative	*E. coli*
		B	negative	*E. coli*
		C	negative	*E. coli*
		D	negative	*E. coli*
	Caecum	A	negative	*E. coli*
		B	negative	*E. coli*
**2**	Doudenum	no growth		
	Small intestine (middle part)	A	negative	*E. coli*
		B	negative	
		C	negative	*E. coli*
		D	negative	*E. coli*
	Small intestine (distal part)	A	negative	*E. coli*
		B	negative	*E. coli*
		C	negative	*E. coli*
		D	negative	*E. coli*
	Caecum	A	negative	*E. coli*
		B	negative	*E. coli*
		C	negative	*E. coli*
		D	negative	*E. coli*
**3**	Doudenum	no growth		
	Small intestine (middle part)	A	negative	
		B	negative	*E. coli*
		C	negative	*E. coli*
		D	negative	*E. coli*
	Small intestine (distal part)	not tested[b]		
	Caecum	not tested[b]		

[a] only the highest identification results with log(score) value above 2.0 were shown
[b] because of overgrowth of bacteria it was not possible to isolate single colonies

Supplementary Table 9. The titer of A5/80 phage in murine blood 60 min after the phage intravenous administration. Mice were given 0.2 ml of the phage lysate diluted with peptone water. Mean results obtained from 2 mice are shown.

Phage dose [pfu/mice]	Phage titer in blood [pfu/ml]
1 000	10
10 000	15
100 000	359
1 000 000	738
10 000 000	4 700

2.2 Supplementary Figure

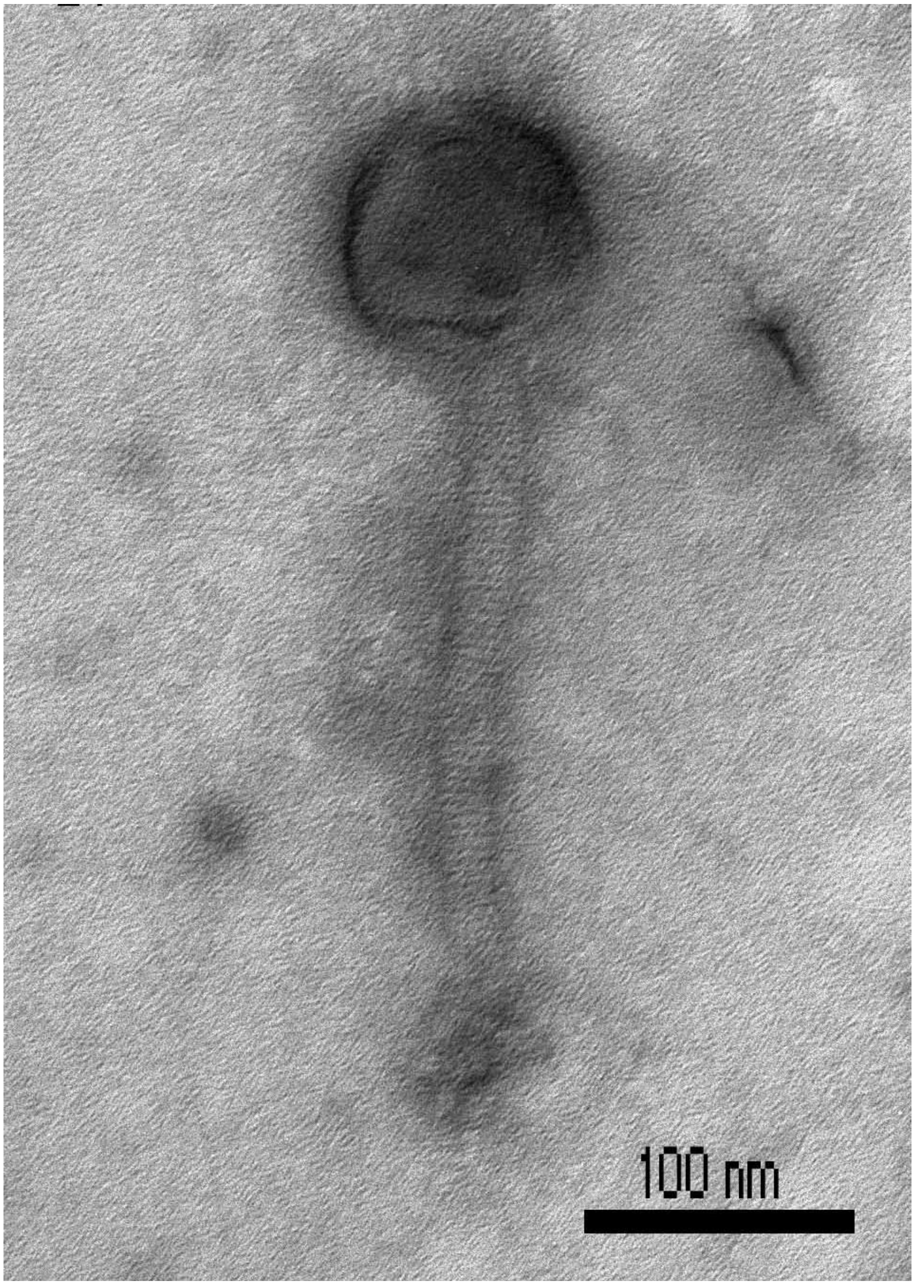

Supplementary Figure 1. Transmition electron micrograph of a therapeutic staphylococcal A5/80 phage from the bacteriophage collection of the Hirszfeld Institute of Immunology and Experimental Therapy, Polish Academy of Sciences in Wroclaw, Poland. We thank dr Jerzy Kassner from the Electron Microscopy Facility of HIIET PAS for providing this picture.

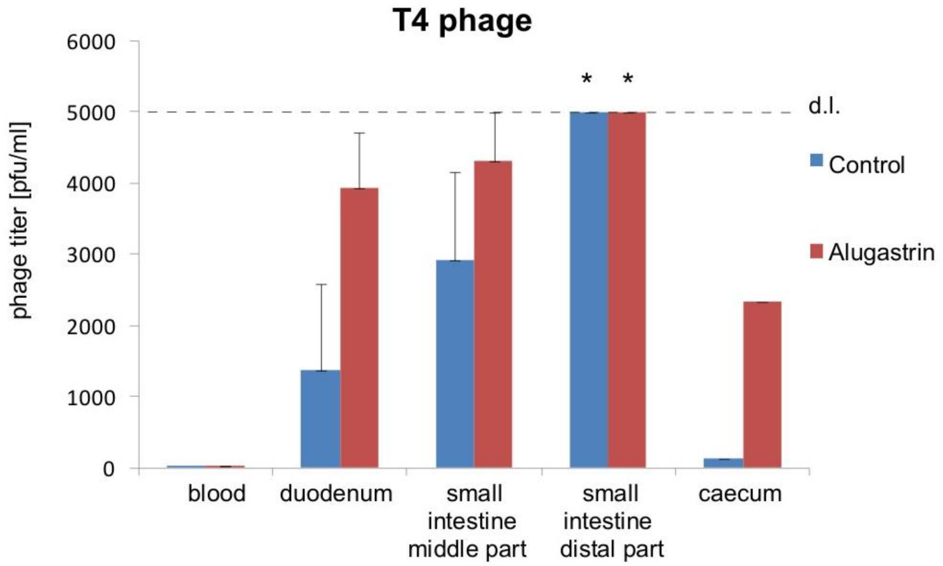

Supplementary Figure 2. Bioavailability of T4 phage (dose: 0.5 ml, 5.8×10^9 pfu per animal) in blood and intestines 60 min after its oral administration to rats. Alugastrin (1.0 ml) was applied 10 min before the phage administration. The mean phage titer ±SE in analyzed samples (n = 4) is shown. Asterisks indicate that the mean value was above the upper detection limit (d.l.) of the phage titer in these experiments.
